# 

**Published:** 1890-02-22

**Authors:** 


### NEW OFFICES IN THE STRAND.

The Hospital *is now published at* **140, Strand, W.C.** *It has been found desirable to establish* The Hospital *in this central and convenient locality, owing to the rapid extension of the business of this paper of late.*

### NOTICE TO CORRESPONDENTS.

All MS., Letters, Books for Review, and other matters intended for the Editor should be addressed The Editor, The Lodge, Porchester Square, London, W.

**Notice to Advertisers and Subscribers.**

All Advertisements, Orders for Copies of The Hospital, and Business Communications should be addressed to The Publisher, *not to the Editor*, The Hospital, 140, Strand, W.C.

**Bound Volumes of "The Hospital."**

Vol. VI., containing full page portraits of T.R.H. the Prince and Princess of Wales, and Miss Florence Nightingale, is now ready, 4s. post free. Cases for binding may be had of the Publisher, at 1s. 6d. each.

**To Residents, Secretaries, and Matrons.**

The Editor will be glad to have the Regulations and each Report as issued by Asylums, Hospitals, Convalescent and Nursing Institutions, as well as those of other Charities, and an early copy in duplicate of all Appeals and Festival Notices, etc., addressed to *The Editor, The Lodge, Porchester Square, London, W.* Any superintendent, secretary, matron, or other chief official who regularly sends such information may be registered, on application to the Publisher, to receive free of cost a cover for binding The Hospital in half-yearly volumes.

### Scraps and Gleanings.

There are fifty women on the medical register in Scotland.

The St. John Ambulance Association is flourishing in Ceylon.

Mr. Thomas White has given £500 to Evesham Cottage Hospital.

The Maternity Hospital of the City Road treated 422 patients last year.

Lord Dudley has opened the Guest Hospital, and given £200 to clear it from debt.

State aid for imbeciles and idiots is likely to be shortly granted. It is badly needed.

The new wing of the Hospital for Sick Children, Great Ormond Street, is really begun.

The Duchess of Portland has passed the third examination in First-aid Ambulance work.

Several hospitals have lately received large cheques from the proprietor of *Tit Bits*.

An International Exhibition of Medical Science will be opened at Berlin next August.

Kenilworth Convalescent Home has reduced its patients' fees from 5s. 6d. to 4s. a-week.

Mr. J. S. A. Walker has been appointed assistant medical officer to Hope Hospital, Manchester.

Dr. C. Theodore Williams has given £500 to Brompton Hospital for Consumption in memory of his father.

Mrs. Oliphant has written an account of the Royal Hospital for Incurables, which she calls a Home of Peace.

Aberdeen Royal Infirmary still exercises commendable economy, and is now able to boast a small balance in hand.

Manchester Consumption Hospital treated 231 in-patients and 5,235 out-patients last year. The hospital is £57 in debt.

The Farningham Boys' Home held their annual conversazione, at the Mansion House, at the invitation of the Lord Mayor.

The house surgeon of Durham County Hospital desires to be addressed as the resident medical officer. What's in a name?

Buchanan Cottage Hospital treated 184 patients last year. The hospital contains 16 beds, and will probably soon be enlarged.

Newcastle Hospital for Chest Diseases is flourishing, but is unable to find a suitable house in which to take up a new abode.

The fifth Parsee lady medical student, Miss Dhunbai Master, took her degree last month at the Grand Medical College, Bombay, with great credit.

The East London Nursing Society will hold their annual meeting at the Mansion House on February 25th, at three o'clock, the Lord Mayor in the chair.

The Goldsmiths' Company have contribued £500 towards the extension and building fund of the proposed new London Opththalmic Hospital, Moorfields.

At the twenty-second annual dinner of the French Hospital and Dispensary on Saturday last, subscriptions amounting to £2,000 were announced.

On February 22nd, at 5 p.m., in the chapel of the Hospital for Sick Children, Great Ormond Street, a special service will be held in memory of the late Mr. Barry.

The committee of the Home of Rest for Horses have made arrangements to transfer the home from Sudbury to more extensive premises at Friars Place Farm, Acton.

Sir Hedworth Williamson opened the new ward and extension of the Monkwearmouth and Southwick Hospital on February 8th, in the presence of a large company.

Those who desire to be octogenarians had better go and live at Shorne, near Gravesend. Six persons have died there this year; their ages were 76, 78, 80, 83, 88, 95; total, 500!

The Archbishop of Paris, exercising the option given by the Pope, in consideration of the influenza epidemic, has dispensed with Lenten fast and abstinence in his diocese up to March 23rd.

Mr. Ewen J. Maclean, M.B., C.M. Edinburgh, has been appointed house surgeon to Bristol Hospital for Sick Children and Women, in succession to Dr. Lockhart Livingstone, resigned.

Mr. William Powell, of Dove Bank Mills, Mellor, near Marple Bridge, has extensive works in course of erection for the manufacture of wadding, cotton-wool, surgical dressings, and specialities at Cataract Bridge, Mellor.

A sermon will be preached in St. Philip's, Regent Street, Waterloo Place, by the Rev. E. C. Hawkins, vicar of St. Bride's, Fleet Street, at four o'clock on Sunday afternoon, March 30th, when the offertory will be devoted to the East London Nursing Society.

The After-care Association for Poor and Friendless Female Convalescents, of which the Princess Christian is patron, and Lord Meath president, exercises a benevolent supervision over persons sent forth from lunatic asylums. Funds are needed for this deserving charity.

The Executive Council of the French Exhibition, which opens at Earl's Court on Saturday, May 3rd, has decided to present a liberal portion of its surplus to the building fund of the new French Hospital in London, and to give one or more fêtes for the benefit of that fund during the six months the exhibition will be open.

Mrs. Ernest Hart has left London for the west coast of Ireland, for the purpose of supervising the technical teaching and developing the organisation of the Donegal Industrial Fund in the villages of that desolate district, where it is stated that the people are eagerly profiting by the teaching and the opportunities for industrial work given by the fund.

